# On reciprocal degree distance of graphs

**DOI:** 10.1016/j.heliyon.2023.e17914

**Published:** 2023-07-04

**Authors:** Mingqiang An, Yinan Zhang, Kinkar Chandra Das, Yilun Shang

**Affiliations:** a College of Science, Tianjin University of Science and Technology, Tianjin, 300457, PR China; b College of Artificial Intelligence, Tianjin University of Science and Technology, Tianjin, 300457, PR China; c Department of Mathematics, Sungkyunkwan University, Suwon 16419, Republic of Korea; d Department of Computer and Information Sciences, Northumbria University, Newcastle, NE1 8ST, UK

**Keywords:** 05C09, 05C92, Reciprocal degree distance, *ħ*-hamiltonian, *ħ*-path-coverable, *ħ*-edge-hamiltonian

## Abstract

Given a connected graph *H*, its reciprocal degree distance is defined as

$$RDD(H)=\sum_{x\neq y}\frac{d_{H}(v_{x})+d_{H}(v_{y})}{d_{H}(v_{x},v_{y})},$$

where $d_{H}(v_{x})$ denotes the degree of the vertex $v_{x}$ in the graph *H* and $d_{H}(v_{x},v_{y})$ is the shortest distance between $v_{x}$ and $v_{y}$ in *H*. The goal of this paper is to establish some sufficient conditions to judge that a graph to be *ħ*-hamiltonian, *ħ*-path-coverable or *ħ*-edge-hamiltonian by employing the reciprocal degree distance.

## 1 Introduction

In this paper, we are only concerned with simple graphs (also known as networks). We assume these graphs are undirected, connected and finite. Let H=(V(H),E(H)) be a connected graph. The vertex set is represented by $V(H)=\{v_{1},\;v_{2},\ldots ,\;v_{p}\}$, and the cardinalities of the vertex set and edge set are |V(H)|=p and |E(H)|=m, respectively. The degree of $v_{x}$ in *H* is represented by $d_{H}(v_{x})\;(=d_{x})$. Let $d_{H}(v_{x},v_{y})$ be the length of a shortest path linking $v_{x}$ to $v_{y}$ in the graph topology *H*. We call the sequence $\pi (H)=(d_{1},d_{2},\ldots ,d_{p})$ the *degree sequence* for the network topology *H*. Throughout this paper we can assume that $d_{1}\leq d_{2}\leq \cdots \leq d_{p}$. If there is no ambiguity, we usually delete footnote *H* from the symbols in the following.

We use $H_{1}+H_{2}$ (resp. $H_{1}\cup H_{2}$) to denote the *join* (resp. *union*) of two graphs $H_{1}$ and $H_{2}$ assuming they do not share any vertices. The complete graph $K_{p}$ represents an all-to-all topology with *p* vertices.

If the subgraph induced by V(H)∖X (X⊆V(H) with |X|≤ħ) is hamiltonian, then we say that the connected graph *H* is *ħ-hamiltonian*. Therefore 0-hamiltonian is similar to that of hamiltonian. A *hamiltonian path* of a graph *H* is nothing but a sequence of incident edges covering exactly all vertices in the graph *H*. At this moment, we call *H traceable*. In general, a graph *H* is *ħ-path-coverable* if we can cover the vertex set by no more than *ħ* paths which do not have any two identical vertices. These paths are called vertex-disjoint in graph theory. Especially, 1-path-coverable is similar to that of traceable. We call a connected graph *H ħ-edge-hamiltonian* (*ħ* is a positive integer) under the following condition: If we choose a group of vertex-disjoint paths which have less than or equal to *ħ* edges totally, the group must form a subgraph of some hamiltonian cycle of *H*. Standard graph theory terminology, see e.g., [1], is used in this paper.

Molecular structure descriptors are also referred to as topological indices. These indices form an important part of the theoretical and mathematical chemistry, which are used to describe the characteristics of chemical compounds [2]. Numerous topological indices have so far been presented and found a wide range of practical applications [3], including the Harary index [4] and Wiener index [5]. Other interesting results could be obtained in [6], [7], [8], [9], [10], [11], [12] and the references therein.

If *H* is connected, then we define the *reciprocal degree distance* RDD(H) in the following expression:

(1) $$RDD(H)=\sum_{i\neq j}\frac{d_{H}(v_{i})+d_{H}(v_{j})}{d_{H}(v_{i},v_{j})}.$$

The reciprocal degree distance was first introduced by Alizadeh, Iranmanesh and Došlić in [13] under the name *additively weighted Harary index* because this invariant is regarded as an additively weighted variant of the Harary index. The present name for RDD(H) was first put forward in the work of Hua and Zhang [14]. In that work, this topological index was independently introduced. We refer the readers to [15], [16], [17], [18], [19], [20] for the latest results related to RDD(H).

Denote ${\overset{˜}{D}}_{H}(x)=\Sigma_{y\in V(H)﹨\{x\}}\frac{1}{d_{H}(y,x)}$. So we can write Eq. (1) in the following new form.

(2) $$RDD(H)=\sum_{x\in V(H)}d_{H}(x){\overset{˜}{D}}_{H}(x).$$

One can easily obtain

(3) $${\overset{˜}{D}}_{H}(x)\leq d(x)+\frac{1}{2}\;(p-1-d(x)).$$

In graph theory, it is often interesting and challenging to determine whether a graph has some kind of important graph property. For example, it is NP-complete to decide whether a given graph is hamiltonian [21]. Until now, plenty of mathematicians have worked on this problem and have got some exciting results. For instance, separately in [22], [23], the authors studied traceability by employing the Harary index or the Wiener index. These results were generalized in [24], [25], [26], [27]. However, as far as we know, there are only few such sufficient conditions based on topological indices.

Motivated by [16], [28], sufficient conditions to judge that a graph to be *ħ*-hamiltonian, *ħ*-path-coverable and *ħ*-edge-hamiltonian by using the reciprocal degree distance are given respectively in this paper. The following theorems are verified.

Theorem 1.1 *Let* $H\;(≇K_{\hbar +1}+(K_{1}\cup K_{p-\hbar -2}\;),\;0\leq \hbar \leq p-3)$ *be a graph with* p≥6 *vertices. If*

(4) $$RDD(H)\geq \frac{1}{2}\;[{\left(p-1\right)}^{2}(2p-\hbar )+\hbar^{2}+(4p-3)\hbar -(p-1)(6p-11)],$$

*then H is ħ-hamiltonian.*

Theorem 1.2 *Let* $H\;(\notin \{K_{1}+(K_{p-\hbar -2}\cup \bar{K_{\hbar +1}}\;),\;\bar{K_{\frac{p+\hbar +1}{2}}}+K_{\frac{p-\hbar -1}{2}},\;K_{\frac{p-\hbar -2}{2}}+(K_{2}\cup \bar{K_{\frac{p+\hbar -2}{2}}}\;)\},\;1\leq \hbar \leq p-3)$ *be a graph with* p≥11 *vertices. Let* $\hbar_{1}=\frac{1}{9}\;(13p-17-2\sqrt{31p^{2}-43p+16})$ *and* $\hbar_{1}^{⁎}=\frac{1}{9}\;(13p-14-2\sqrt{31p^{2}-55p+22})$ *be two integers.*

(*i*) *For* p−ħ−1 *is even and* $\hbar \in [1,\hbar_{1}]$*, or* p−ħ−1 *is odd and* $\hbar \in [1,\hbar_{1}^{⁎}]$ *or* ħ=p−4*, if*

(5) $$RDD(H)\geq \frac{1}{2}(p-\hbar -1)(p-\hbar )(2p-\hbar -2)-2\hbar^{2}+(6p-7)\hbar -\frac{5}{2}p^{2}+\frac{15}{2}p-5,$$

*then H is ħ-path-coverable.*

(ii) *For* p−ħ−1 *is even and* $\hbar \in (\hbar_{1},p-3)$ *, if*

(6) $$RDD(H)\geq \frac{1}{16}\;[8\;(p-\hbar -1)(p-\hbar )(2p-\hbar -2)+9\hbar^{3}-(35p-29)\hbar^{2}+(31p^{2}-46p+15)\hbar -5p^{3}+5p^{2}+5p-5],$$

*then H is ħ-path-coverable.*

(iii) *For* p−ħ−1 *is odd and* $\hbar \in (\hbar_{1}^{⁎},p-4)$ *, if*

(7) $$RDD(H)\geq \frac{1}{16}\;[8\;(p-\hbar -1)(p-\hbar )(2p-\hbar -2)+9\hbar^{3}-(35p-32)\hbar^{2}+(31p^{2}-52p+12)\hbar -5p^{3}-4p^{2}+44p-32],$$

*then H is ħ-path-coverable.*

Theorem 1.3 *Let* $H\;(≇K_{\hbar +1}+(K_{1}\cup K_{p-\hbar -2}),\;0\leq \hbar \leq p-3)$ *be a graph with* p≥6 *vertices. If*

(8) $$RDD(H)\geq \frac{1}{2}\;[(p+\hbar -1)\;(p+\hbar )(2p+\hbar -2)-\hbar^{3}-(4p-4)\hbar^{2}-(5p^{2}-11p+5)\hbar -5p^{2}+15p-10],$$

*then H is ħ-edge-hamiltonian.*

The paper is constructed as follows. In the next section we establish lemmas that will be invoked in the following section. Section 3 contains the theoretical derivation for Theorem 1.1, Theorem 1.2, Theorem 1.3.

## 2 Preliminaries

In this section, we give some lemmas which will be used later.

Lemma 2.1 [16] *Let* $\nu (t)=(p-1)t+t^{2}$ *for* 1≤t≤p−1 *. Then* ν(t) *is a strictly increasing function of t.*

Lemma 2.2 *Let* $\pi =(t_{1}\leq t_{2}\leq \cdots \leq t_{p})$ *be a graphical sequence,* 0≤ħ≤p−3 *.*

(*a*) [29] *If*

$$t_{k}\leq k+\hbar \Rightarrow t_{p-k-\hbar }\geq p-k\;\text{for}\;1\leq k<\frac{1}{2}(p-\hbar ),$$

*then π enforces ħ-hamiltonian.*

(*b*) [30] *If*

$$t_{k-\hbar }\leq k\Rightarrow t_{p-k}\geq p-k+\hbar \;\text{for}\;\hbar +1\leq k<\frac{1}{2}(p+\hbar ),$$

*then π enforces ħ-edge-hamiltonian.*

Lemma 2.3 [31], [32] *Let* $\pi =(t_{1}\leq t_{2}\leq \cdots \leq t_{p})$ *be a graphical sequence,* ħ≥1 *. If*

$$t_{k+\hbar }\leq k\Rightarrow t_{p-k}\geq p-k-\hbar \;\text{for}\;1\leq k<\frac{1}{2}(p-\hbar ),$$

*then π enforces ħ-path-coverable.*

## 3 Proofs of main results

Now, we prove Theorem 1.1, Theorem 1.2, Theorem 1.3.

Proof of Theorem 1.1 Assume to the contrary that *H* is not *ħ*-hamiltonian. Then by Lemma 2.2 (*a*), there exists an integer $1\leq k<\frac{1}{2}(p-\hbar )$ such that $d_{k}\leq k+\hbar$ and $d_{p-k-\hbar }\leq p-k-1$. We have 0≤ħ≤p−3. Then from Lemma 2.1, Eq. (2) and inequality (3), we obtain

$$RDD(H)=\sum_{x\in V(H)}d(x)\overset{˜}{D}(x)\leq \sum_{x\in V(H)}d(x)(d(x)+\frac{1}{2}(p-1-d(x)))=\frac{1}{2}\;((p-1)\sum_{x\in V(H)}d(x)+\sum_{x\in V(H)}d{\left(x\right)}^{2})\leq \frac{1}{2}(p-1)\;[k(k+\hbar )+(p-2k-\hbar )(p-1-k)+(k+\hbar )(p-1)]\;\;\;\;\;\;\;\;\;\;\;\;\;+\frac{1}{2}[k{\left(k+\hbar \right)}^{2}+(p-2k-\hbar ){\left(p-k-1\right)}^{2}+(k+\hbar ){\left(p-1\right)}^{2}]=\frac{1}{2}{\left(p-1\right)}^{2}(2p-\hbar )-\frac{1}{2}\;[k^{3}-(8p+\hbar -7)k^{2}+((p-1)(6p-4\hbar -3)-\hbar^{2})k].$$

We define

$$f(x)=x^{3}-(8p+\hbar -7)x^{2}+((p-1)(6p-4\hbar -3)-\hbar^{2})x\;\;\text{ with }1\leq x\leq \frac{1}{2}(p-\hbar -1).$$

Then we have

$$f^{\prime }(x)=3x^{2}-2(8p+\hbar -7)x+(p-1)(6p-4\hbar -3)-\hbar^{2},\;\text{ and }\;f^{\prime \prime }(x)=6x-2(8p+\hbar -7).$$

Since p≥6, $x\leq \frac{1}{2}(p-\hbar -1)$ and ħ≥0, we obtain

$$f^{\prime \prime }(x)=3\;[2x-(p-\hbar -1)]-13p-5\hbar +11\leq -13p+11<0.$$

Hence f(x) is a strictly concave up function for $1\leq x\leq \frac{p-\hbar -1}{2}$. Since *x* is an integer, we have

**Case 1.**p−ħ−1 is even. Then $f(x)\geq \text{min}\{f(1),\;f(\frac{p-\hbar -1}{2})\}$. Direct calculations yield

(9) $$f(1)=-\hbar^{2}-(4p-3)\hbar +(p-1)(6p-11),$$

and

$$f(\frac{p-\hbar -1}{2})=\frac{1}{8}(\hbar^{3}+(3p-5)\hbar^{2}-(p-1)(13p-5)\hbar +(9p+1){\left(p-1\right)}^{2}).$$

After subtraction,

$$f(\frac{p-\hbar -1}{2})-f(1)=\frac{1}{8}\;(\hbar^{3}+(3p+3)\hbar^{2}-(13p^{2}-50p+29)\hbar +9p^{3}-65p^{2}+143p-87).$$

We define

$$\Theta_{1}(\hbar )=\hbar^{3}+(3p+3)\hbar^{2}-(13p^{2}-50p+29)\hbar +9p^{3}-65p^{2}+143p-87\;\text{ for }\hbar \in [0,\;p-3].$$

Let $\hbar_{1}^{1}\leq \hbar_{2}^{1}\leq \hbar_{3}^{1}$ be the three roots of the equation $\Theta_{1}(\hbar )=0$. Using Matlab, we get that the three solutions, respectively, are

$$\hbar_{1}^{1}=-2p-\sqrt{13p^{2}-38p+29},\;\hbar_{2}^{1}=p-3\;\text{ and }\hbar_{3}^{1}=-2p+\sqrt{13p^{2}-38p+29}.$$

Since p≥6, we have $\hbar_{1}^{1}<0$, $\Theta_{1}(0)=9p^{3}-65p^{2}+143p-87>0$ and $13p^{2}-38p+29>{\left(3p-2\right)}^{2}$. So $\hbar_{3}^{1}>-2p+\sqrt{{\left(3p-2\right)}^{2}}=p-2>p-3=\hbar_{2}^{1}$. Since the function $\Theta_{{}_{1}}(\hbar )$ is continuous in the interval [0,p−3], we have $\Theta_{1}(\hbar )\geq 0$ for ħ∈[0,p−3]. This implies that $f(\frac{p-\hbar -1}{2})-f(1)\geq 0$. Therefore f(x)≥f(1). Hence

$$RDD(H)\leq \frac{1}{2}\;[{\left(p-1\right)}^{2}(2p-\hbar )+\hbar^{2}+(4p-3)\hbar -(p-1)(6p-11)].$$

Combining inequality (4) with the above result, we conclude that

$$RDD(H)=\frac{1}{2}\;[{\left(p-1\right)}^{2}(2p-\hbar )+\hbar^{2}+(4p-3)\hbar -(p-1)(6p-11)].$$

Thus we have k=1, and hence $d_{1}=\hbar +1$, $d_{2}=\cdots =d_{p-\hbar -1}=p-2$, $d_{p-\hbar }=\cdots =d_{p}=p-1$. Therefore $H≅K_{\hbar +1}+(K_{1}\cup K_{p-\hbar -2}\;)$, which contradicts the assumption. So *H* is *ħ*-hamiltonian.

**Case 2.**p−ħ−1 is odd. Then the minimum value of f(x) can only be obtained in f(1) and $f(\frac{p-\hbar -2}{2})$. Since ħ≠p−3, we have ħ∈[0,p−4]. Direct calculations result in

$$f(\frac{p-\hbar -2}{2})=\frac{1}{8}(\hbar^{3}+(3p-8)\hbar^{2}-(13p^{2}-12p+8)\hbar +9p^{3}-12p^{2}-24p+24).$$

By subtracting Eq. (9),

$$f(\frac{p-\hbar -2}{2})-f(1)=\frac{1}{8}\;(\hbar^{3}+3p\hbar^{2}-(13p^{2}-44p+32)\hbar +9p^{3}-60p^{2}+112p-64).$$

We now define

$$\Theta_{2}(\hbar )=\hbar^{3}+3p\hbar^{2}-(13p^{2}-44p+32)\hbar +9p^{3}-60p^{2}+112p-64\;\text{ for }\hbar \in [0,\;p-4].$$

Let $\hbar_{1}^{2}\leq \hbar_{2}^{2}\leq \hbar_{3}^{2}$ be the three roots of the equation $\Theta_{2}(\hbar )=0$. Calculated by Matlab, we get that the three solutions, respectively, are

$$\hbar_{1}^{2}=2-2p-\sqrt{13p^{2}-32p+20},\;\hbar_{2}^{2}=p-4\;\text{ and }\hbar_{3}^{2}=2-2p+\sqrt{13p^{2}-32p+20}.$$

Since p≥6, we obtain $\hbar_{1}^{2}<0$, $\Theta_{2}(0)=9p^{3}-60p^{2}+112p-64>0$ and $13p^{2}-32p+20>{\left(3p-5\right)}^{2}$. Hence $\hbar_{3}^{2}>2-2p+\sqrt{{\left(3p-5\right)}^{2}}=p-3>p-4=\hbar_{2}^{2}$. Since the function $\Theta_{2}(\hbar )$ is continuous in [0,p−4], we obtain $\Theta_{2}(\hbar )\geq 0$ for ħ∈[0,p−4], which means that $f(\frac{p-\hbar -2}{2})-f(1)\geq 0$. Therefore f(x)≥f(1). Thus the remaining proof is the same as the proof in **Case 1**, and we are done for this case. □

Proof of Theorem 1.2 Assume to the contrary that *H* is not *ħ*-path-coverable. Therefore according to Lemma 2.3, there is an integer *k* with $k\in [1,\;\frac{1}{2}(p-\hbar -1)]$ satisfying that $d_{k+\hbar }\leq k$ and $d_{p-k}\leq p-\hbar -k-1$. Recall that 1≤ħ≤p−3. So from Lemma 2.1, Eq. (2) and inequality (3), we obtain

$$RDD(H)\leq \frac{1}{2}((p-1)\sum_{x\in V(H)}d(x)+\sum_{x\in V(H)}d{\left(x\right)}^{2})\leq \frac{1}{2}(p-1)\;[(\hbar +k)k+(p-\hbar -2k)(p-\hbar -k-1)+(p-1)k]\;\;\;\;\;\;\;\;\;\;\;\;\;\;\;\;+\frac{1}{2}[(\hbar +k)k^{2}+(p-\hbar -2k){\left(p-\hbar -k-1\right)}^{2}+{\left(p-1\right)}^{2}k]=\frac{1}{2}\;(p-1-\hbar )(p-\hbar )(2p-\hbar -2)-\frac{1}{2}[k^{3}-(8p-4\hbar -7)k^{2}\;\;\;\;\;\;\;\;\;\;\;\;\;\;\;\;\;+((p-4\hbar )\;(p-1)+2(2p-2\hbar -1)\;(p-\hbar -1))\;k].$$

Let us consider a function

$$g(x)=x^{3}-(8p-4\hbar -7)x^{2}+((p-1)(p-4\hbar )+2(2p-2\hbar -1)\;(p-\hbar -1))x$$

for $1\leq x\leq \frac{1}{2}(p-\hbar -1)$, 1≤ħ≤p−3. We obtain

$$g^{\prime }(x)=3x^{2}-2(8p-4\hbar -7)x+(p-4\hbar )\;(p-1)+2(p-\hbar -1)(2p-2\hbar -1),\;\text{ and }\;g^{\prime \prime }(x)=6x-2(8p-4\hbar -7).$$

Since p≥11, $x\leq \frac{1}{2}(p-\hbar -1)$ and ħ≤p−3, we obtain

$$g^{\prime \prime }(x)=3\;[2x-(p-\hbar -1)]-13p+5\hbar +11\leq -8p-4<0.$$

This implies that g(x) is a strictly concave function on $x\in [1,\frac{p-\hbar -1}{2}]$. Recall that *x* is an integer, we obtain

**Case 1.**p−ħ−1 is even. Then $g(x)\geq \text{min}\{g(1),\;g(\frac{p-\hbar -1}{2})\}$. By direct calculation, we get

(10) $$g(1)=4\hbar^{2}-(12p-14)\hbar +5p^{2}-15p+10,$$

$$g(\frac{p-\hbar -1}{2})=\frac{1}{8}(-9\hbar^{3}+(35p-29)\hbar^{2}-(31p^{2}-46p+15)\hbar +5p^{3}-5p^{2}-5p+5).$$

After subtraction,

$$g(\frac{p-\hbar -1}{2})-g(1)=\frac{1}{8}(-9\hbar^{3}+(35p-61)\hbar^{2}-(31p^{2}-142p+127)\hbar +5p^{3}-45p^{2}+115p-75).$$

We define

$$\Gamma_{1}(\hbar )=-9\hbar^{3}+(35p-61)\hbar^{2}-(31p^{2}-142p+127)\hbar +5p^{3}-45p^{2}+115p-75\;\text{ for }\hbar \in [1,\;p-3].$$

It is not difficult to verify that $\hbar_{1}=\frac{1}{9}\;(13p-17-2\sqrt{31p^{2}-43p+16})$ is a root of the equation $\Gamma_{1}(\hbar )=0$. Suppose that the equation $\Gamma_{1}(\hbar )=0$ has two other roots, says $\hbar_{2}$ and $\hbar_{3}$. Without loss of generality, we may assume that $\hbar_{2}\leq \hbar_{3}$. With the aid of Matlab, we can get that the two distinct solutions, respectively, are $\hbar_{2}=p-3$ and $\hbar_{3}=\frac{1}{9}(13p-17+2\sqrt{31p^{2}-43p+16})$.

Since p≥11, we get $\hbar_{1}>1$, $\hbar_{3}>p-3$, $\Gamma_{1}(1)=5p^{3}-76p^{2}+292p-272>0$ and $31p^{2}-43p+16>{\left(2p+5\right)}^{2}$. Therefore $\hbar_{1}<\frac{1}{9}(13p-17-2(2p+5))=p-3=\hbar_{2}$. Note that the function $\Gamma_{1}(\hbar )$ is continuous in the interval [1,p−3].

**Subcase 1.1.**$\hbar \in [1,\hbar_{1}]$. Then $\Gamma_{1}(\hbar )\geq 0\Rightarrow g(\frac{p-\hbar -1}{2})\geq g(1)$. Hence g(1)≤g(x). Thus

$$RDD(H)\leq \frac{1}{2}(p-1-\hbar )(p-\hbar )(2p-\hbar -2)-2\hbar^{2}+(6p-7)\hbar -\frac{5}{2}p^{2}+\frac{15}{2}p-5.$$

In combination with inequality (5), the above inequality can only be true if we take equal sign. Thus k=1, and hence $d_{1}=d_{2}=\cdots =d_{\hbar +1}=1$, $d_{\hbar +2}=\cdots =d_{p-1}=p-\hbar -2$ and $d_{p}=p-1$. So $H≅K_{1}+(K_{p-\hbar -2}\cup \bar{K_{\hbar +1}}\;)$, a contradiction. Hence *H* is *ħ*-path-coverable.

**Subcase 1.2.**$\hbar \in (\hbar_{1},p-3)$. Then $\Gamma_{2}(\hbar )<0$. It implies that $g(\frac{p-\hbar -1}{2})-g(1)<0$. Therefore $g(x)\geq g(\frac{p-\hbar -1}{2})$. Thus we have

$$RDD(H)\leq \frac{1}{16}\;[8\;(p-\hbar -1)(p-\hbar )(2p-\hbar -2)+9\hbar^{3}-(35p-29)\hbar^{2}\;\;\;\;\;\;\;\;\;\;\;\;\;\;\;\;\;\;\;\;\;\;\;+(31p^{2}-46p+15)\hbar -5p^{3}+5p^{2}+5p-5].$$

Combining inequality (6) with the above result, we obtain

$$RDD(H)=\frac{1}{16}\;[8(p-\hbar -1)\;(p-\hbar )(2p-\hbar -2)+9\hbar^{3}-(35p-29)\hbar^{2}+(31p^{2}-46p+15)\hbar -5p^{3}+5p^{2}+5p-5].$$

Thus we have $k=\frac{p-\hbar -1}{2}$, and hence $d_{1}=d_{2}=\cdots =d_{\frac{p+\hbar +1}{2}}=\frac{p-\hbar -1}{2}$, $d_{\frac{p+\hbar +3}{2}}=\cdots =d_{p}=p-1$. So $H≅\bar{K_{\frac{p+\hbar +1}{2}}}+K_{\frac{p-\hbar -1}{2}}$, a contradiction. Hence *H* is *ħ*-path-coverable.

**Case 2.**p−ħ−1 is odd. Then the minimum value of g(x) can only be obtained in g(1) and $g(\frac{p-\hbar -2}{2})$. Since ħ≠p−3, we have ħ∈[1,p−4]. Note that

$$g(\frac{p-\hbar -2}{2})=\frac{1}{8}(-9\hbar^{3}+(35p-32)\hbar^{2}-(31p^{2}-52p+12)\hbar +5p^{3}+4p^{2}-44p+32).$$

By subtracting Eq. (10),

$$g(\frac{p-\hbar -2}{2})-g(1)=\frac{1}{8}(-9\hbar^{3}+(35p-64)\hbar^{2}-(31p^{2}-148p+124)\hbar +5p^{3}-36p^{2}+76p-48).$$

Define

$$\Gamma_{2}(\hbar )=-9\hbar^{3}+(35p-64)\hbar^{2}-(31p^{2}-148p+124)\hbar +5p^{3}-36p^{2}+76p-48\;\text{ for }\hbar \in [1,\;p-4].$$

It is not difficult to check that $\hbar_{1}^{⁎}=\frac{1}{9}(13p-14-2\sqrt{31p^{2}-55p+22})$ is a root of the equation $\Gamma_{2}(\hbar )=0$. Suppose that the equation $\Gamma_{2}(\hbar )=0$ has two other roots, says $\hbar_{2}^{⁎}$ and $\hbar_{3}^{⁎}$ such that $\hbar_{2}^{⁎}\leq \hbar_{3}^{⁎}$. Using Matlab, one can easily see that the two distinct solutions, respectively, are $\hbar_{2}^{⁎}=p-4$ and $\hbar_{3}^{⁎}=\frac{1}{9}\;(13p-14+2\sqrt{31p^{2}-55p+22})$.

Since p≥11, we have $\hbar_{1}^{⁎}>1$, $\hbar_{3}^{⁎}>p+3$, $\Gamma_{2}(1)=5p^{3}-67p^{2}+259p-245>0$ and $31p^{2}-55p+22>{\left(2p+11\right)}^{2}$. Thus $\hbar_{1}^{⁎}<\frac{1}{9}(13p-14-2(2p+11))=p-4=\hbar_{2}^{⁎}$. The function $\Gamma_{2}(\hbar )$ is continuous in [1,p−4]. We now consider the following two subcases.

**Subcase 2.1.**ħ=p−4 or $\hbar \in [1,\hbar_{1}^{⁎}]$. Thus $\Gamma_{2}(\hbar )\geq 0$, which means that $g(\frac{p-\hbar -2}{2})\geq g(1)$. So g(1)≤g(x). Hence the remaining proof is the same as the proof in **Subcase 1.1**, and we are done for this case.

**Subcase 2.2.**$\hbar \in (\hbar_{1}^{⁎},p-4)$. Then $\Gamma_{2}(\hbar )<0$. It implies that $g(\frac{p-\hbar -2}{2})-g(1)<0$. Therefore $g(x)\geq g(\frac{p-\hbar -2}{2})$. Thus

$$RDD(H)\leq \frac{1}{16}[8(p-\hbar -1)(p-\hbar )(2p-\hbar -2)+9\hbar^{3}-(35p-32)\hbar^{2}\;\;\;\;\;\;\;\;\;\;\;\;\;\;\;\;\;\;\;\;\;\;\;\;\;+(31p^{2}-52p+12)\hbar -5p^{3}-4p^{2}+44p-32].$$

Combining inequality (7) with the above result, we obtain

$$RDD(H)=\frac{1}{16}[8(p-\hbar -1)(p-\hbar )(2p-\hbar -2)+9\hbar^{3}-(35p-32)\hbar^{2}+(31p^{2}-52p+12)\hbar -5p^{3}-4p^{2}+44p-32].$$

Thus $k=\frac{p-\hbar -2}{2}$, and correspondingly $d_{1}=d_{2}=\cdots =d_{\frac{p+\hbar -2}{2}}=\frac{p-\hbar -2}{2}$, $d_{\frac{p+\hbar }{2}}=d_{\frac{p+\hbar +2}{2}}=\frac{p-\hbar }{2}$, $d_{\frac{p+\hbar +4}{2}}=\cdots =d_{p}=p-1$. So $H≅K_{\frac{p-\hbar -2}{2}}+(K_{2}\cup \bar{K_{\frac{p+\hbar -2}{2}}}\;)$, a contradiction. Hence *H* is *ħ*-path-coverable. □

Proof of Theorem 1.3 Assume to the contrary that *H* is not *ħ*-edge-hamiltonian. Thus by Lemma 2.2 (*b*), there exists an integer $\hbar +1\leq k\leq \frac{1}{2}(p+\hbar -1)$ such that $d_{k-\hbar }\leq k$ and $d_{p-k}\leq p+\hbar -k-1$. Note that 0≤ħ≤p−3. Therefore from Lemma 2.1, Eq. (2) and inequality (3), we obtain

$$RDD(H)\leq \frac{1}{2}((p-1)\sum_{x\in V(H)}d(x)+\sum_{x\in V(H)}d{\left(x\right)}^{2})\leq \frac{1}{2}(p-1)[(k-\hbar )k+(p-2k+\hbar )(p+\hbar -k-1)+k(p-1)]+\frac{1}{2}[(k-\hbar )k^{2}+(p-2k+\hbar ){\left(p+\hbar -k-1\right)}^{2}+k{\left(p-1\right)}^{2}]=\frac{1}{2}\;(p+\hbar -1)\;(p+\hbar )(2p+\hbar -2)-\frac{1}{2}\;[k^{3}-(8p+4\hbar -7)k^{2}+((p+4\hbar )(p-1)+2(p-1+\hbar )(2p-1+2\hbar ))k].$$

Denote

$$h(x)=x^{3}-(8p+4\hbar -7)x^{2}+((p-1)\;(p+4\hbar )+2(p+\hbar -1)(2p+2\hbar -1))x\;\text{ for }\hbar +1\leq x\leq \frac{1}{2}(p+\hbar -1).$$

Then we have

$$h^{\prime }(x)=3x^{2}-2(8p+4\hbar -7)x+(4\hbar +p)\;(p-1)+2(p-1+\hbar )(2p-1+2\hbar )\;\text{ and }h^{\prime \prime }(x)=6x-2(8p+4\hbar -7).$$

Since p≥6, $x\leq \frac{1}{2}(p+\hbar -1)$ and ħ≥0, we obtain

$$h^{\prime \prime }(x)=3\;[2x-(p+\hbar -1)]-13p-5\hbar +11\leq -13p+11<0.$$

So h(x) is strictly concave up in $\hbar +1\leq x\leq \frac{p+\hbar -1}{2}$. Since *x* is an integer, we have

**Case 1.**p+ħ−1 is even. Then $h(x)\geq \text{min}\{h(\hbar +1),\;h(\frac{p+\hbar -1}{2})\}$. Clearly, ħ≠p−4 in this case. By direct calculation, we obtain

(11) $$h(\hbar +1)=\hbar^{3}+(4p-4)\hbar^{2}+(5p^{2}-11p+5)\hbar +5p^{2}-15p+10,$$

$$h(\frac{p+\hbar -1}{2})=\frac{1}{8}(9\hbar^{3}+(35p-29)\hbar^{2}+(31p^{2}-46p+15)\hbar +5p^{3}-5p^{2}-5p+5).$$

After subtraction,

$$h(\frac{p+\hbar -1}{2})-h(\hbar +1)=\frac{1}{8}(\hbar^{3}+(3p+3)\hbar^{2}-(9p^{2}-42p+25)\hbar +5p^{3}-45p^{2}+115p-75).$$

Define

$$\Omega_{1}(\hbar )=\hbar^{3}+(3p+3)\hbar^{2}-(9p^{2}-42p+25)\hbar +5p^{3}-45p^{2}+115p-75\;\text{ for }\hbar \in [0,\;p-3].$$

Let $\hbar_{1}^{3}\leq \hbar_{2}^{3}\leq \hbar_{3}^{3}$ be the three roots of the equation $\Omega_{1}(\hbar )=0$. With the aid of Matlab, one can easily obtain that the three solutions, respectively, are $\hbar_{1}^{3}=5-5p$, $\hbar_{2}^{3}=p-5$ and $\hbar_{3}^{3}=p-3$.

Since p≥6, we have $\hbar_{1}^{3}<0$ and $\Omega_{1}(0)=5p^{3}-45p^{2}+115p-75>0$. Since the function $\Omega_{1}(\hbar )$ is continuous in [0,p−5], we obtain $\Omega_{1}(\hbar )\geq 0$ for ħ=p−3 or ħ∈[0,p−5]. This implies that $h(\frac{p+\hbar -1}{2})-h(\hbar +1)\geq 0$. Hence h(x)≥h(ħ+1). Thus,

$$RDD(H)\leq \frac{1}{2}\;[(p+\hbar -1)\;(p+\hbar )(2p+\hbar -2)-\hbar^{3}-(4p-4)\hbar^{2}-(5p^{2}-11p+5)\hbar -5p^{2}+15p-10].$$

Combining inequality (8) with the above result, we obtain

$$RDD(H)=\frac{1}{2}\;[(p-1+\hbar )\;(\hbar +p)(2p+\hbar -2)-\hbar^{3}-(4p-4)\hbar^{2}-(5p^{2}-11p+5)\hbar -5p^{2}+15p-10].$$

Thus we have k=1+ħ, and hence $d_{1}=1+\hbar$, $d_{2}=\cdots =d_{p-1-\hbar }=p-2$, $d_{p-\hbar }=\cdots =d_{p}=p-1$. Therefore $H≅K_{\hbar +1}+(K_{1}\cup K_{p-\hbar -2})$, which contradicts the assumption. So *H* is *ħ*-edge-hamiltonian.

**Case 2.**p+ħ−1 is odd. Then the minimum value of h(x) can only be obtained in h(ħ+1) and $h(\frac{p+\hbar -2}{2})$. In this case ħ≠p−3. Note that

$$h(\frac{p+\hbar -2}{2})=\frac{1}{8}(9\hbar^{3}+(35p-32)\hbar^{2}+(31p^{2}-52p+12)\hbar +5p^{3}+4p^{2}-44p+32).$$

By subtracting Eq. (11),

$$h(\frac{p+\hbar -2}{2})-h(\hbar +1)=\frac{1}{8}(\hbar^{3}+3p\hbar^{2}-(9p^{2}-36p+28)\hbar +5p^{3}-36p^{2}+76p-48).$$

Define

$$\Omega_{2}(\hbar )=\hbar^{3}+3p\hbar^{2}-(9p^{2}-36p+28)\hbar +5p^{3}-36p^{2}+76p-48\;\text{ for }\hbar \in [0,\;p-4].$$

Let $\hbar_{1}^{4}\leq \hbar_{2}^{4}\leq \hbar_{3}^{4}$ be the three roots of the equation $\Omega_{2}(\hbar )=0$. By Matlab, one can easily obtain that the three solutions, respectively, are $\hbar_{1}^{4}=6-5p$, $\hbar_{2}^{4}=p-4$ and $\hbar_{3}^{4}=p-2$.

Since p≥6, we get $\hbar_{1}^{4}<0$ and $\Omega_{2}(0)=5p^{3}-36p^{2}+76p-48>0$. Clearly, the function $\Omega_{2}(\hbar )$ is continuous in [0,p−4]. Thus $\Omega_{2}(\hbar )\geq 0$ for ħ∈[0,p−4], that is, $h(\frac{p+\hbar -2}{2})-h(\hbar +1)\geq 0$. Therefore h(x)≥h(ħ+1). Then the rest of the proof is identical to **Case 1**, and we are done for this case. □

## 4 Discussion

In the previous relevant research literature, [15] mainly discussed hamiltonian-connectedness of graphs, and [7], [10] mainly considered two other types of topological indices–the first Zagreb index and the forgotten index. In terms of the reciprocal degree distance (RDD), the *k*-connectivity and *β*-deficiency of graphs are mainly studied in [16]. However, it was unknown to judge graphs to have other properties, such as *ħ*-hamiltonian, *ħ*-path-coverable or *ħ*-edge-hamiltonian by means of RDD. In this paper, these problems are completely solved. Compared with the previous research, we improved the proof method and assisted matlab to solve the problems.

## CRediT authorship contribution statement

Mingqiang An, Yinan Zhang: Conceived and designed the experiments; Analyzed and interpreted the data; Contributed reagents, materials, analysis tools or data; Wrote the paper. Kinkar Chandra Das, Yilun Shang: Performed the experiments; Analyzed and interpreted the data; Wrote the paper

## Declaration of Competing Interest

The authors declare that they have no known competing financial interests or personal relationships that could have appeared to influence the work reported in this paper.

## Data Availability

No data was used for the research described in the article.
